# Supplementary material for: Targeting bacterial nickel transport with aspergillomarasmine A suppresses virulence-associated Ni-dependent enzymes
Source: Nat Commun. 2024 May 13;15:4036. doi: 10.1038/s41467-024-48232-1 (PMC11091072; doi:10.1038/s41467-024-48232-1)
Supplement: Supplementary file 1 — Supplementary Information [file 41467_2024_48232_MOESM1_ESM.pdf]

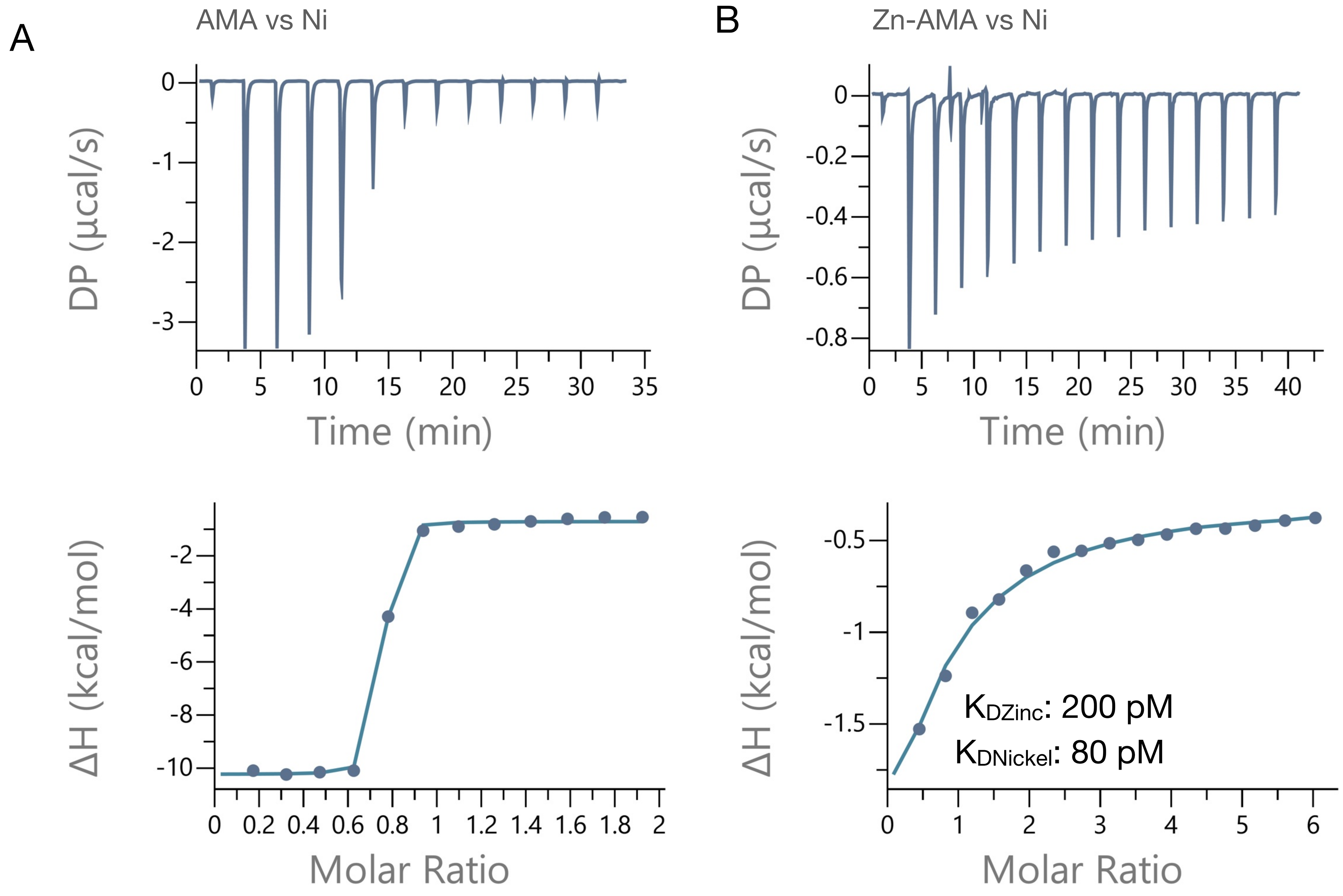

FIG S1 Isothermal titration calorimetry of AMA and Ni^{2+}. (A) Titration of $NiCl_2$ (1 mM) into AMA (0.1 mM) in 25 mM Tris-HCl, 150 mM NaCl, pH 7.5 at 37 ºC. (B) Competitive titration of $NiCl_2$ (1 mM) into a mixture of Zn^{2+} (0.2 mM) and AMA (0.1 mM). The DP value in the y-axis represents differential power. Data were fit using a competitive model.

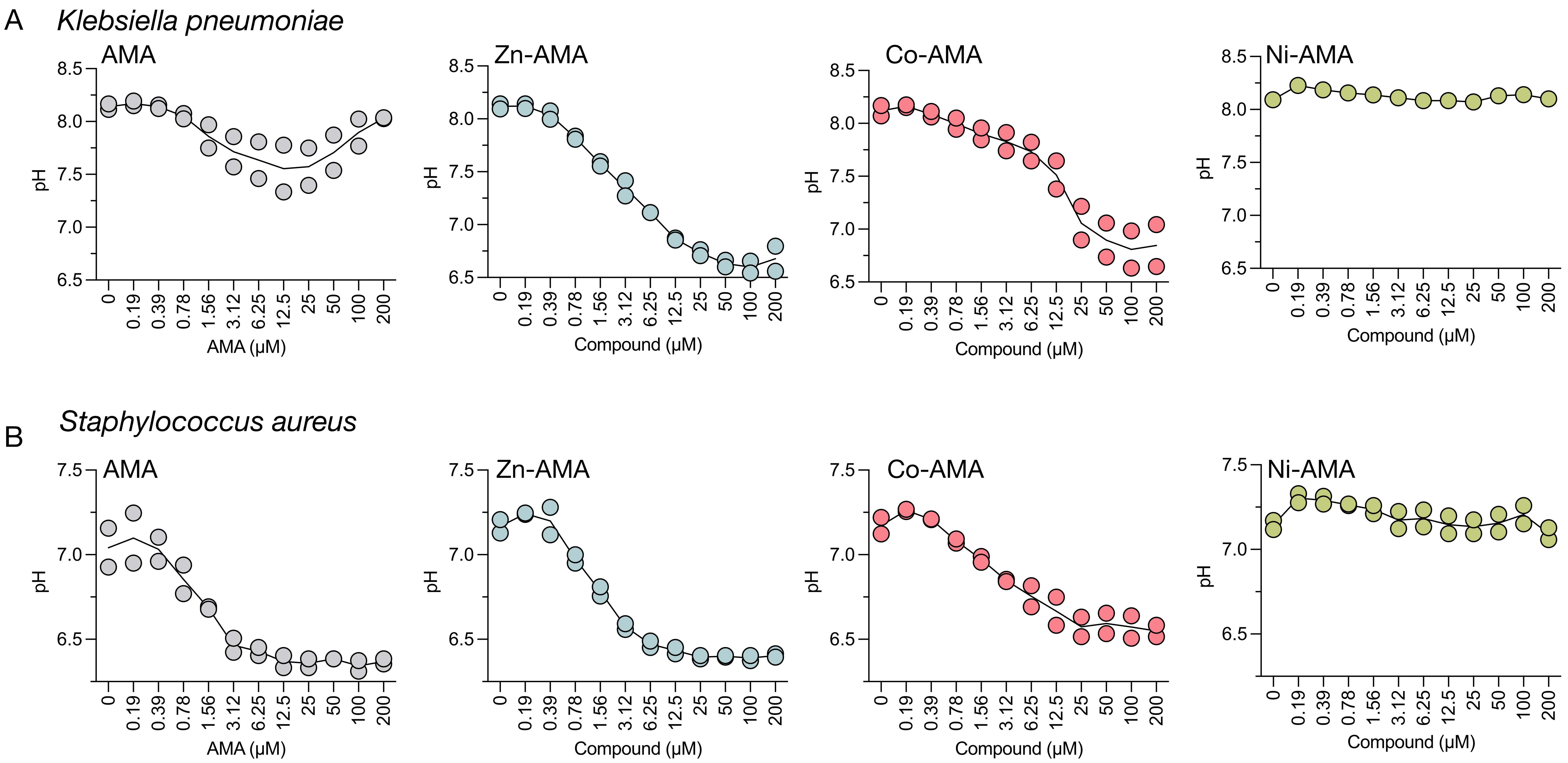

FIG S2 Anti-urease activities of AMA, Zn-AMA, Co-AMA, and Ni-AMA in whole cell assays. Dose-response curves of AMA and metal complexes of AMA in artificial urine inoculated with (A) *K. pneumoniae* ATCC 33495, or (B) *S. aureus* ATCC 29213. Following 24 h of growth at 37 ºC, the pH of the medium was determined using phenol red. Experiments were performed in duplicate. Individual replicates are shown as colored circles with means connected with a black line.

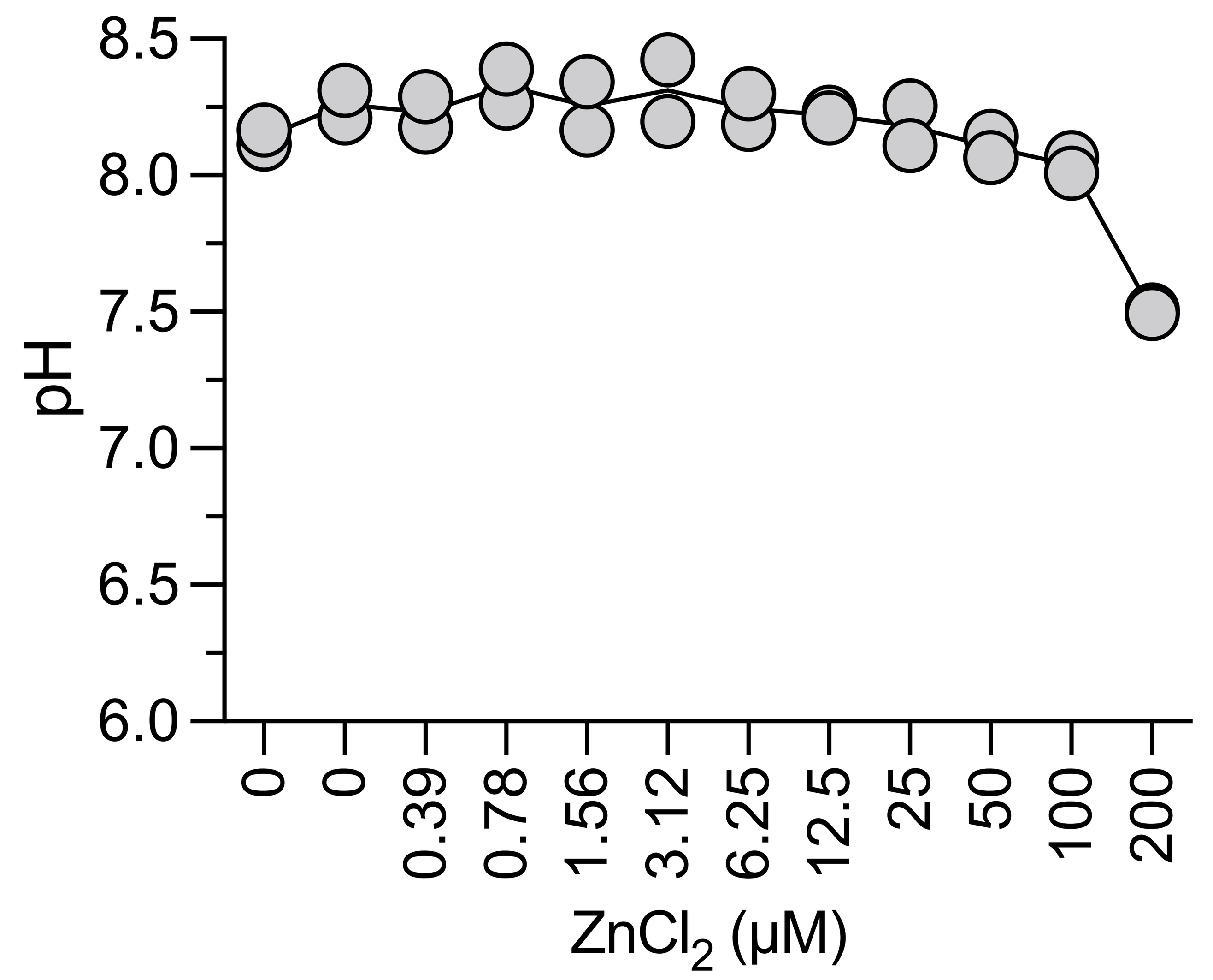

FIG S3 The effect of $ZnCl_2$ on urease activity in whole cells of *K. pneumoniae.* Two-fold dilutions of $ZnCl_2$ were added to artificial urine, inoculated with *K. pneumoniae,* and grown for 24 h. Following 24 h of growth at 37 ºC, the pH of the medium was determined using phenol red. Experiments were performed in duplicate. Individual replicates are shown as colored circles with means connected with a black line.

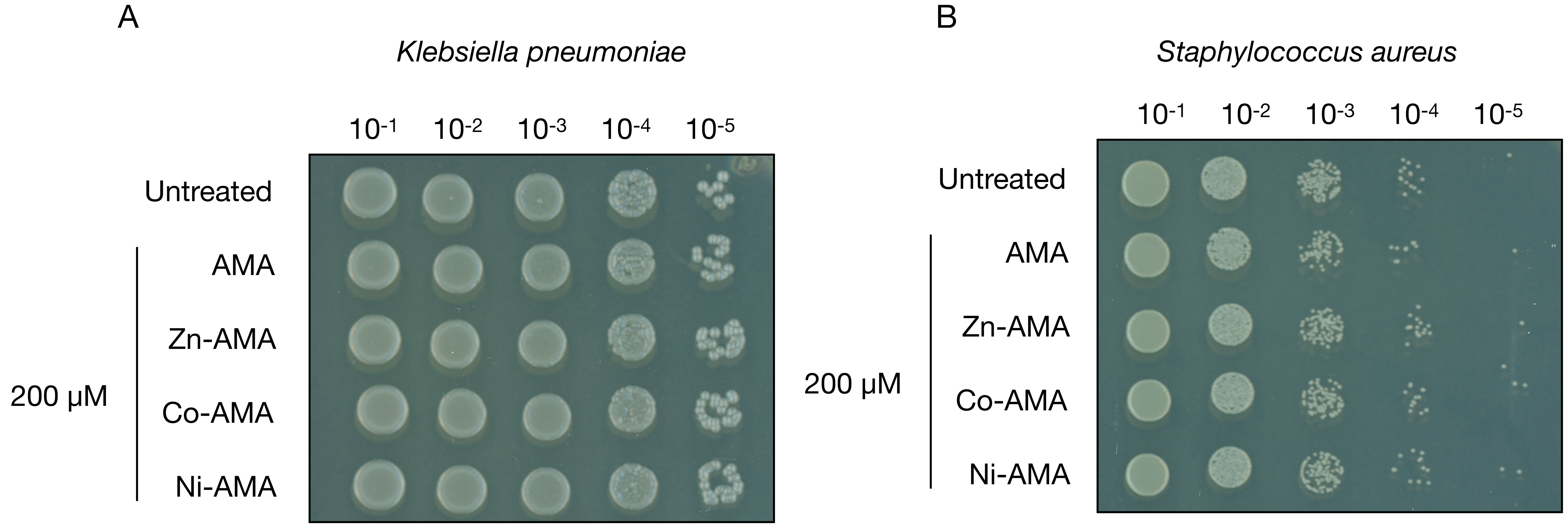

FIG S4 AMA, Zn-AMA, Co-AMA, and Ni-AMA are not lethal to bacteria when grown in artificial urine. Spot dilutions of (A) *K. pneumoniae* and (B) *S. aureus* were grown in artificial urine in the presence of each compound at37 ºC. Following incubation for 24 h, 10-fold spot dilutions were performed on LB agar and grown overnight at 37 ºC.

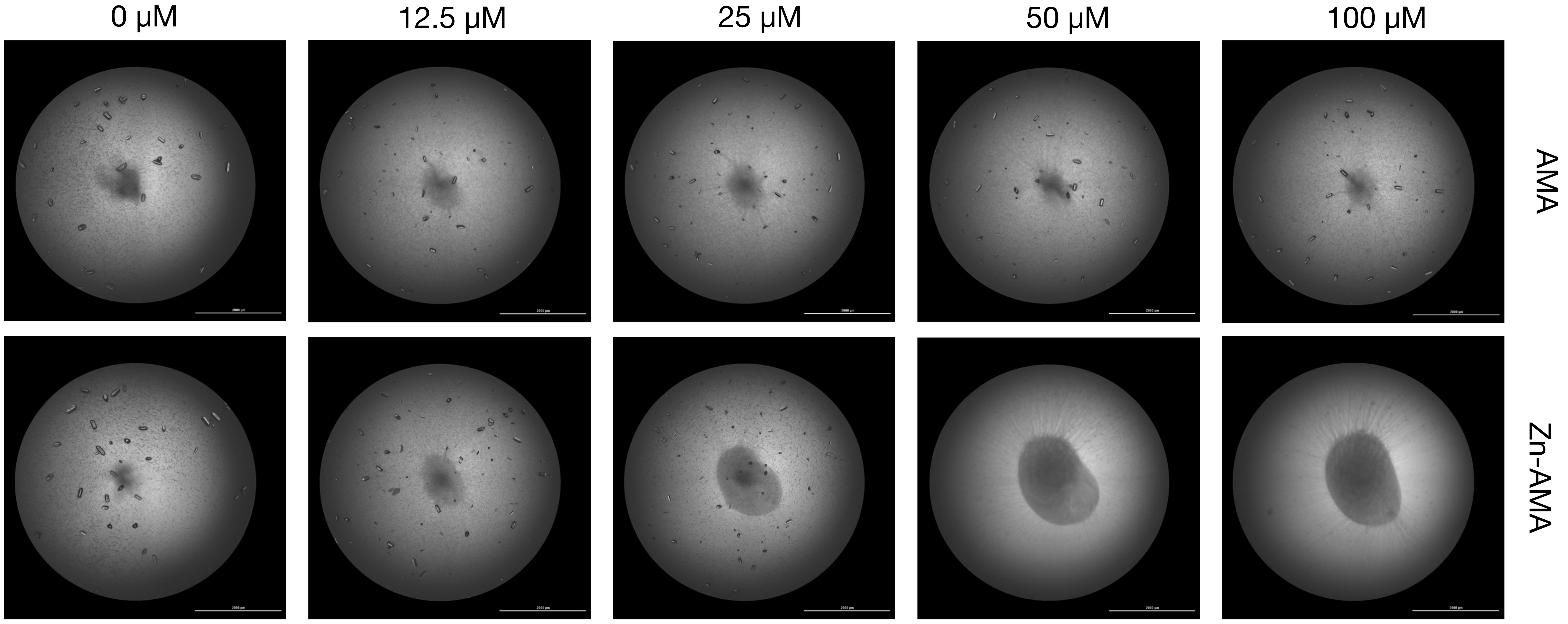

FIG S5 Microscopic detection of struvite crystals grown in artificial urine cultures of *K. pneumoniae.* Top row, the concentration-dependent effect of AMA on struvite formation. Bottom row, the concentration-dependent effect of Zn-AMA on struvite formation. Bacteria were grown in 96 well flat-bottom plates for 24h at 37ºC. Brightfield micrographs were taken with a BioTek Cytation cell imaging reader at 4x magnification. Four images were taken per well and were digitally stitched to generate whole-well images.

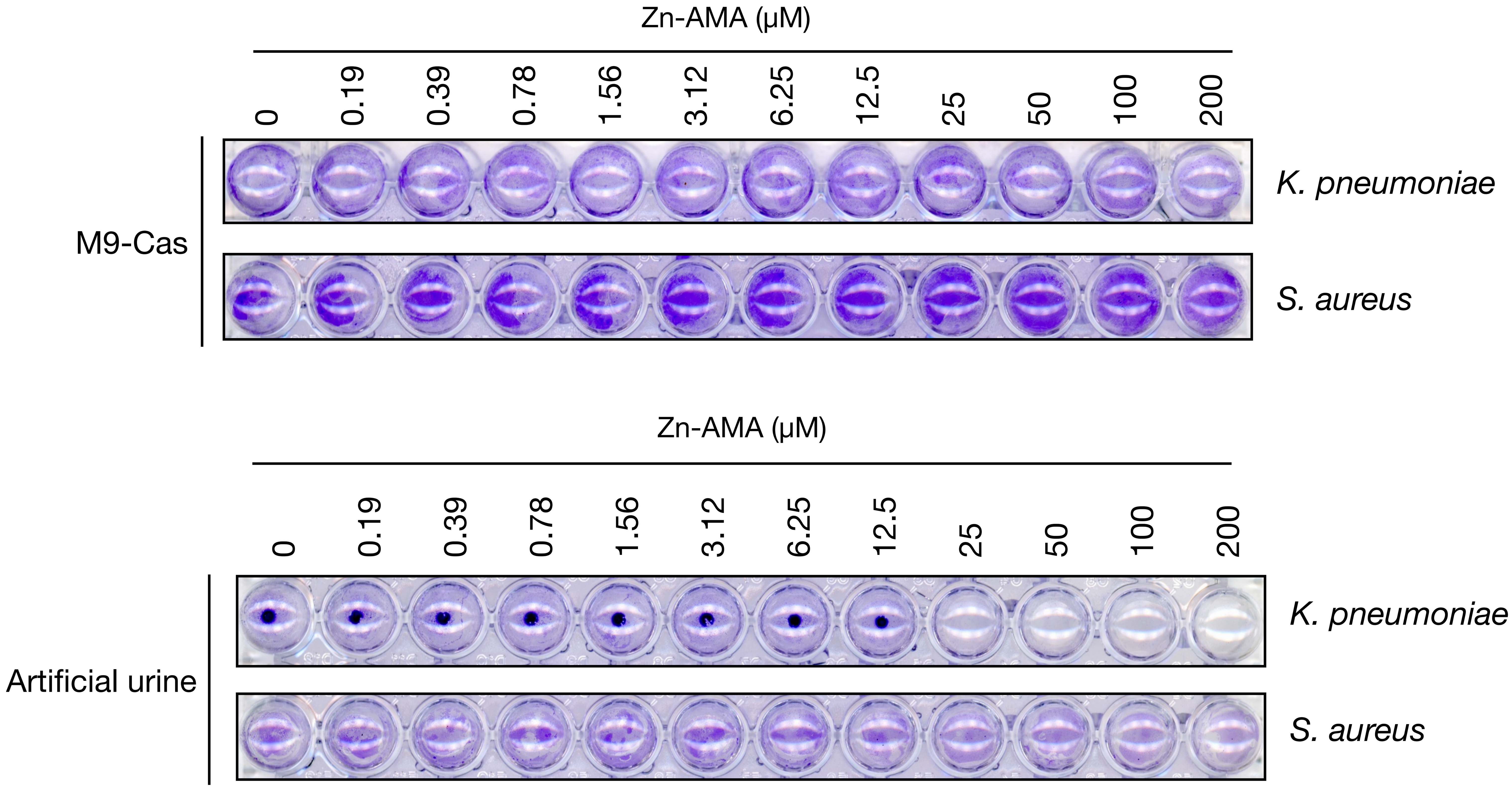

FIG S6 The effect of Zn-AMA on biofilm formation in polystyrene 96-well round-bottom plates. Crystal violet-stained *K. pneumoniae*, and *S. aureus* biofilms from cells grown in M9 minimal medium supplemented with 0.2% casamino acids (M9-Cas; top panel) or artificial urine (bottom panel). Cultures were grown with varying concentrations of Zn-AMA at 37 ºC for 18h and washed three times with water before crystal violet staining.

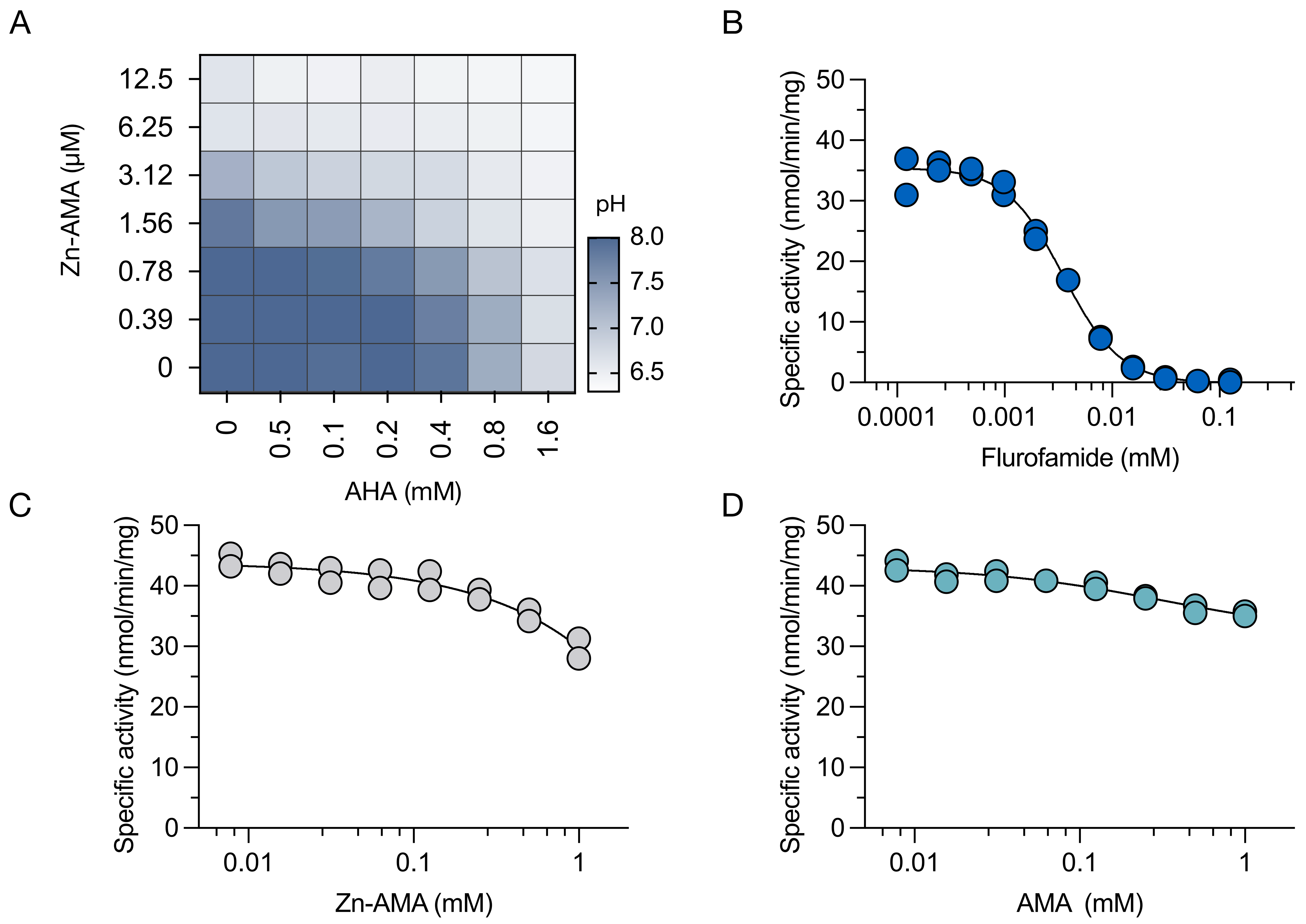

FIG S7 Evaluation of Zn-AMA as a direct inhibitor of urease. A) Checkerboard analysis of Zn-AMA and AHA on urease activity of *K. pneumoniae* after growth for 24 h at 37 ºC artificial urine. pH is represented by the color scale determined with phenol red. **B-D)** The effects of fluorfamide, Zn-AMA, and AMA on the urease-catalyzed release of NH_3 from urea in cell-free extracts of *K. pneumoniae.* Dose-response curves of fluorofamide, Zn-AMA, and AMA inhibiting the urease-catalyzed release of NH_3 quantified with indophenol hypochlorite. The rates of NH_3 production were measured over 20 min and were normalized to total protein concentration. Experiments were performed in duplicate. Individual replicates are shown as colored circles.

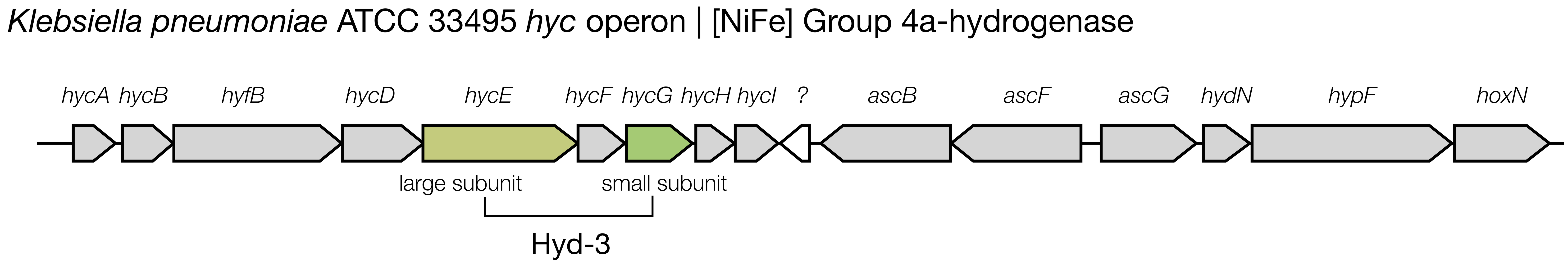

FIG S8 Genetic organization of the *hyc* operon from *K. pneumoniae.* Open reading frames (ORFs) are shown as arrows. The ORFs coloured in green represent the large and small subunits of the Hyd-3 [NiFe] hydrogenase.

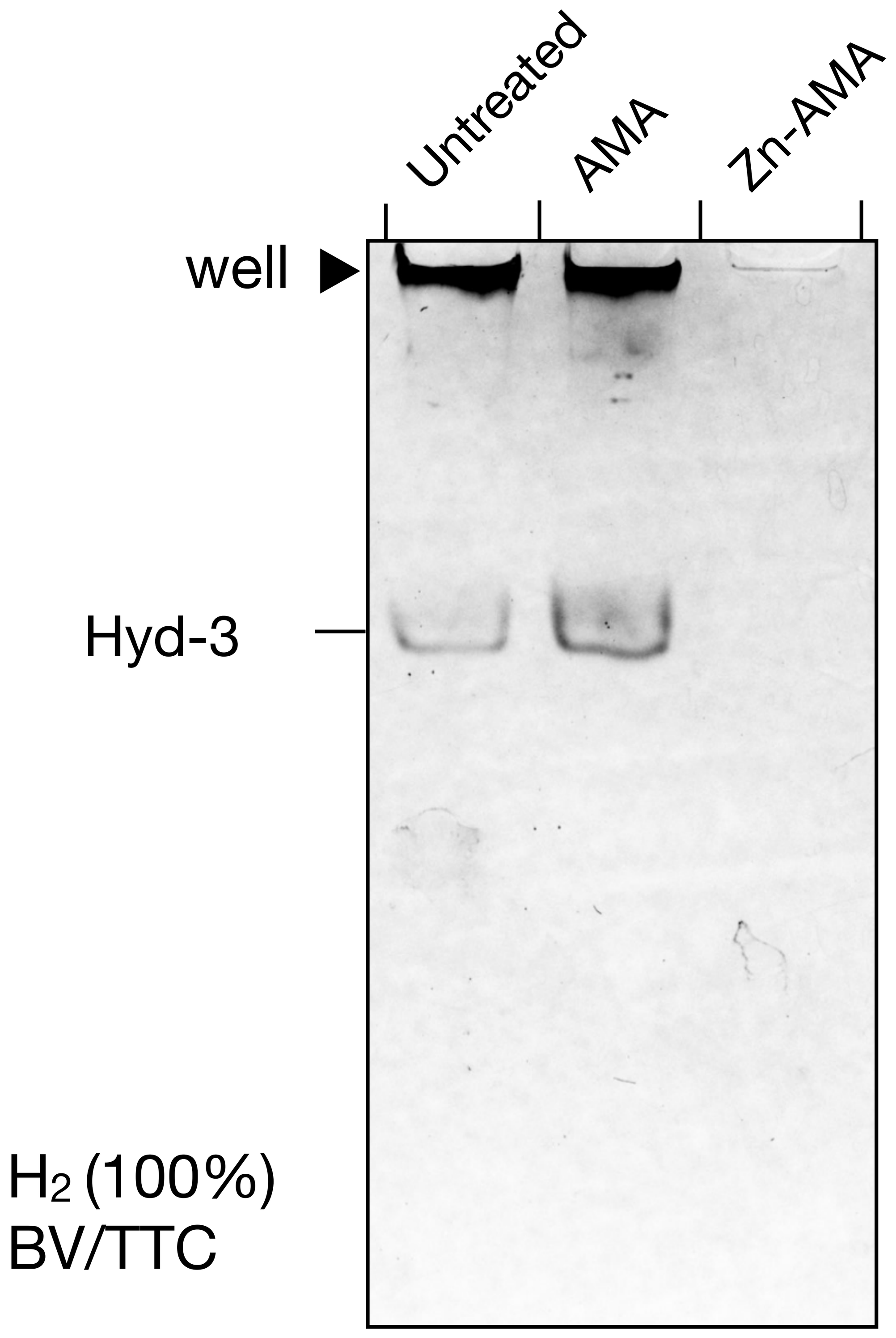

FIG S9 Suppression of Hyd-3 activity by Zn-AMA in *E. coli* detected by zymography. [NiFe] hydrogenase zymograms were performed using *E. coli* grown in LB-glucose supplemented with 0.5 µM $NiCl_2$ for 24h at 37ºC under anaerobic conditions. The *E. coli* was grown in the presence of AMA (150 µM), Zn-AMA (150 µM), or was untreated. Triton X-100 (5% v/v) solubilized membranes were separated using a 12% native PAGE gel containing 0.1% Triton X-100, and bands were developed in 50 mM MOPS pH 7.0 containing 0.5 mg/mL benzyl viologen and 1 mg/mL tetrazolium chloride under 100% H_2 gas.

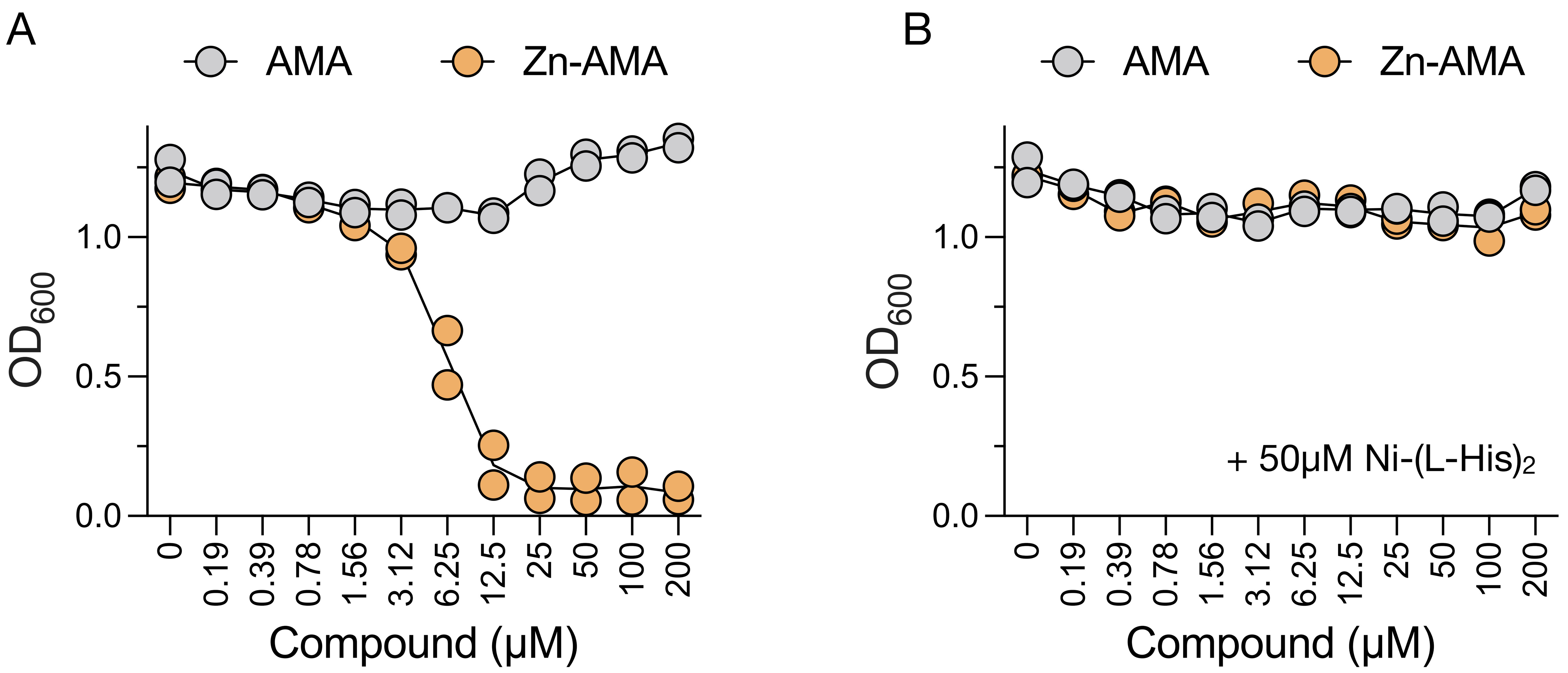

FIG S10 Growth of *K. pneumoniae* in a minimal medium with urea as a sole nitrogen source is inhibited by Zn-AMA. (A) Dose-response curves of AMA and Zn-AMA in minimal medium inoculated with *K. pneumoniae.* (B) Excess Ni-(L-His)$_2$ rescues *K. pneumoniae* from the dose-dependent effect of Zn-AMA. Individual replicates are shown as colored circles.

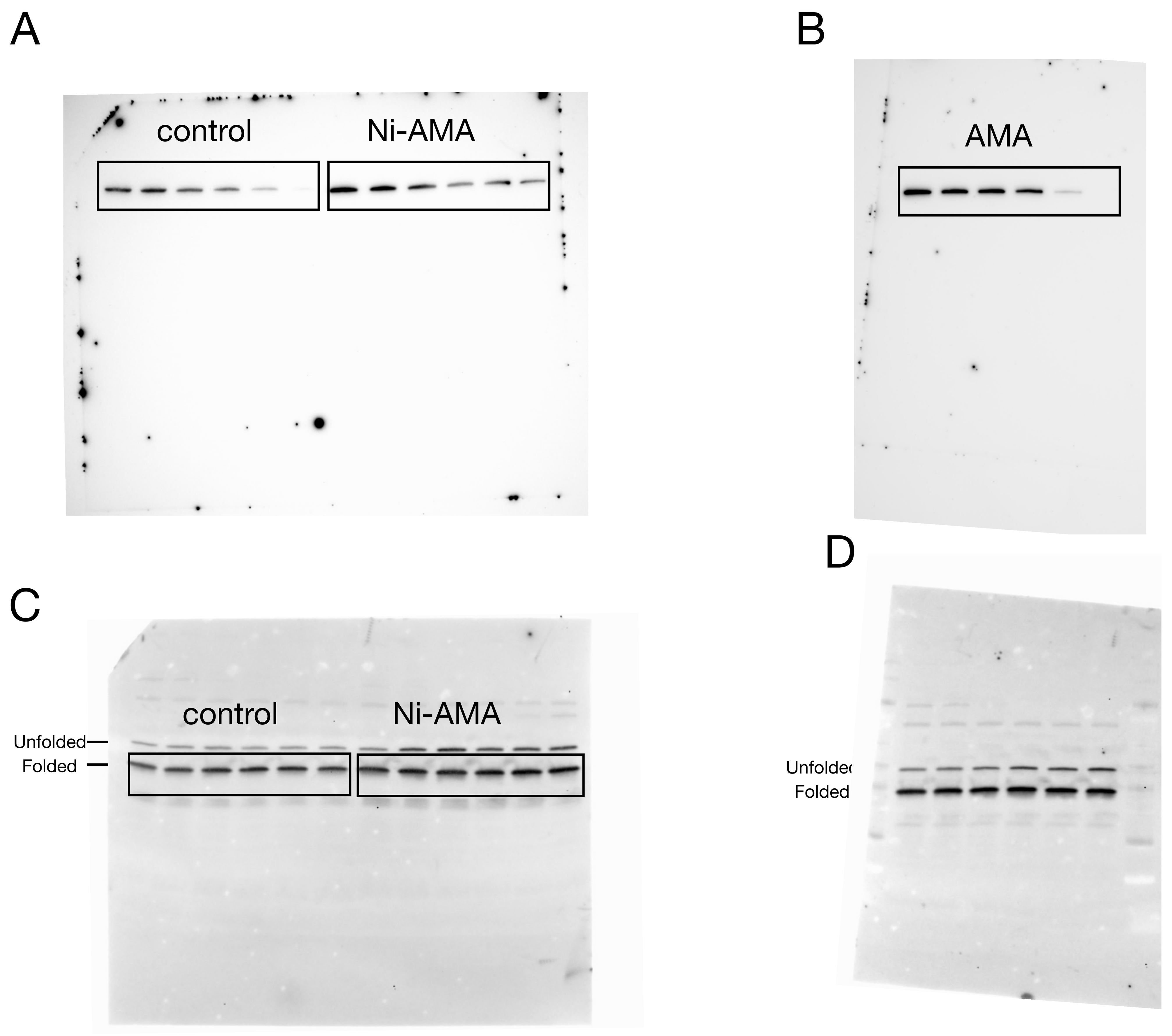

FIG S11 Uncropped western immunoblots from CETSA assays shown in Fig. 2D. (A-B) anti-FLAG blots of (A) control and Ni-AMA treated *K. pneumoniae.* (C-D) corresponding anti-OmpA loading control blots

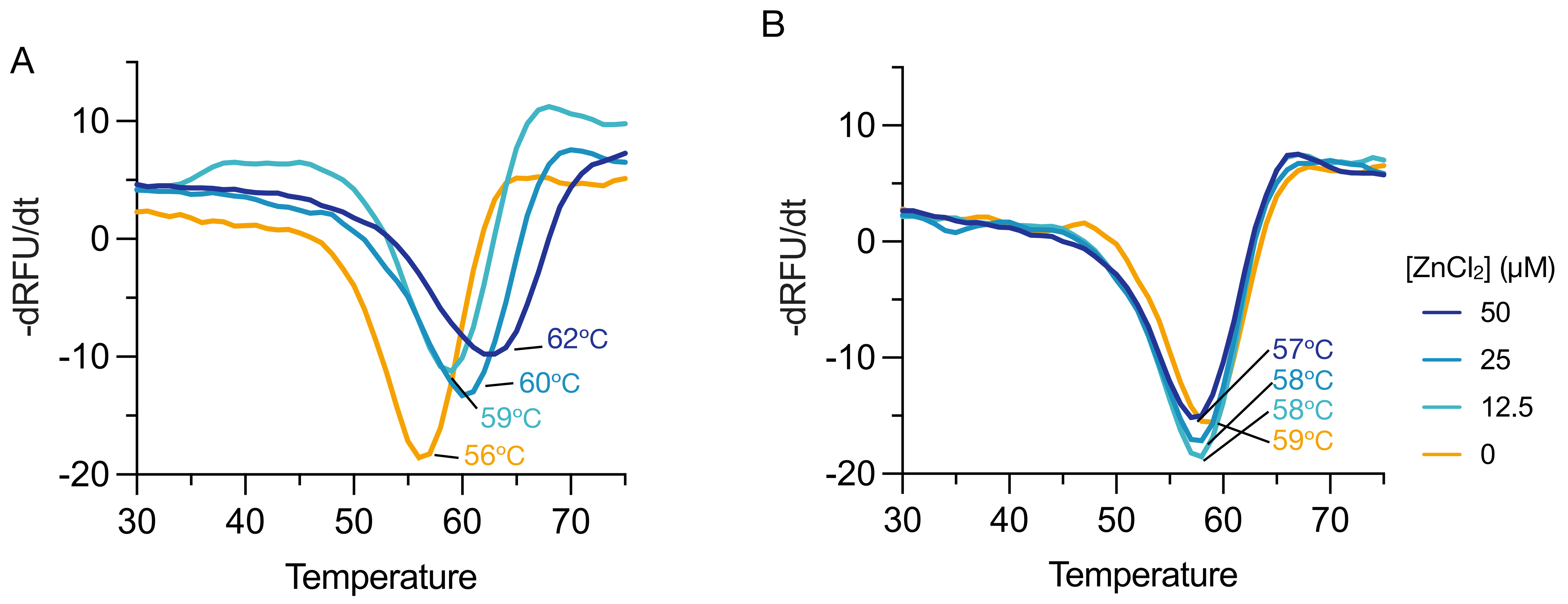

FIG S12 ZnCl$_2$ binding analysis of NikA using thermal shift analysis. Varying amounts of ZnCl$_2$ were added to NikA (2 μM) alone (A), or pre-incubated with Ni-AMA (100 μM) (B) with 2× SYPRO orange. The thermal unfolding of NikA was measured with fluorescence detection, and the y-axis is displayed as negative derivative (-dRFU/dt) plots of the raw output.

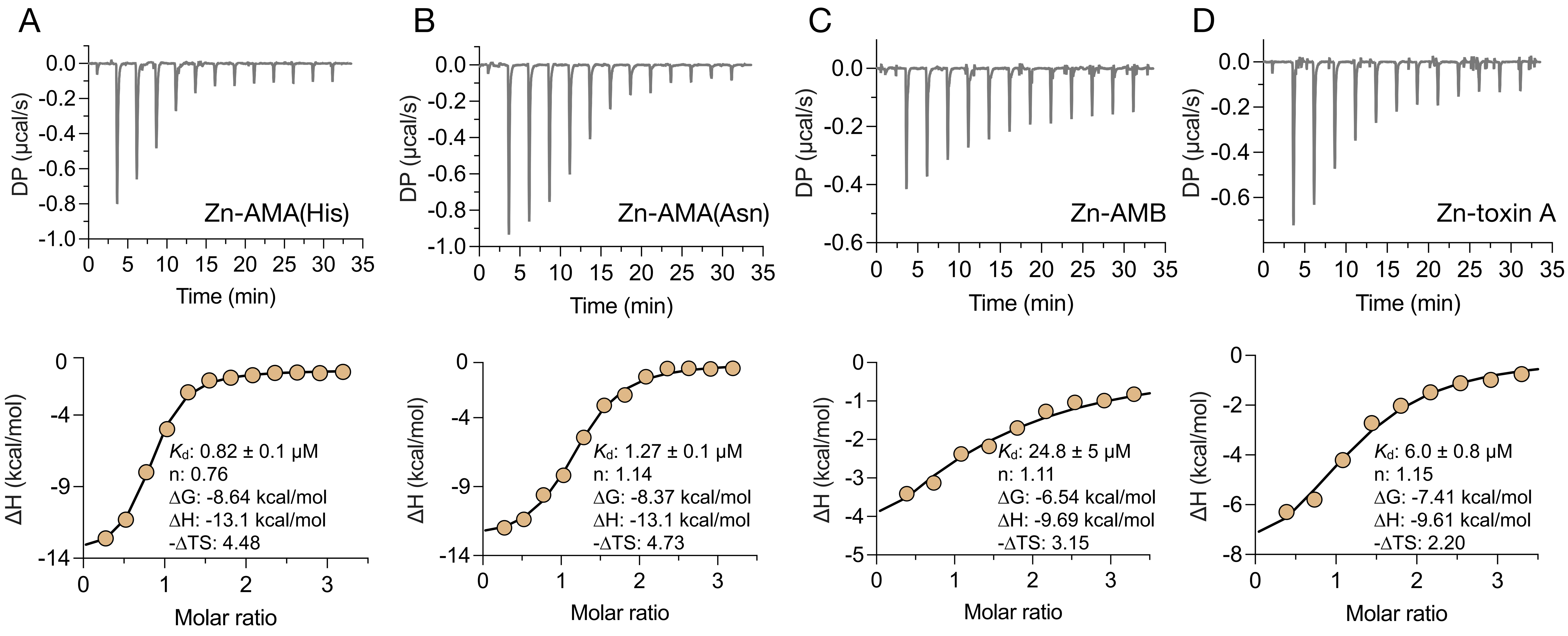

FIG S13 Structure-activity relationships of AMA analogs toward *Kp*NikA. Isothermal titration calorimetry of Zn^{2+} complex AMA analogs with *K. pneumoniae* NikA. Each analog was complexed with ZnCl$_2$ at 1:1 molar ratios, and all titrations were performed were performed in 25 mM Tris-HCl, 150 mM NaCl, 0.1% (v/v) triton X-100, pH 7.5 at 37 ºC. (A) Titration of Zn-AMA(His) (0.25 mM) into *K. pneumoniae* NikA (15 μM). (B) Titration of Zn-AMA(Asn) (0.25 mM) into *K. pneumoniae* NikA (15 μM). (C) Titration of AMB (0.35 mM) into *K. pneumoniae* NikA (15 μM). (D) Zn-ToxinA (0.35 mM) into *K. pneumoniae* NikA (15 μM).

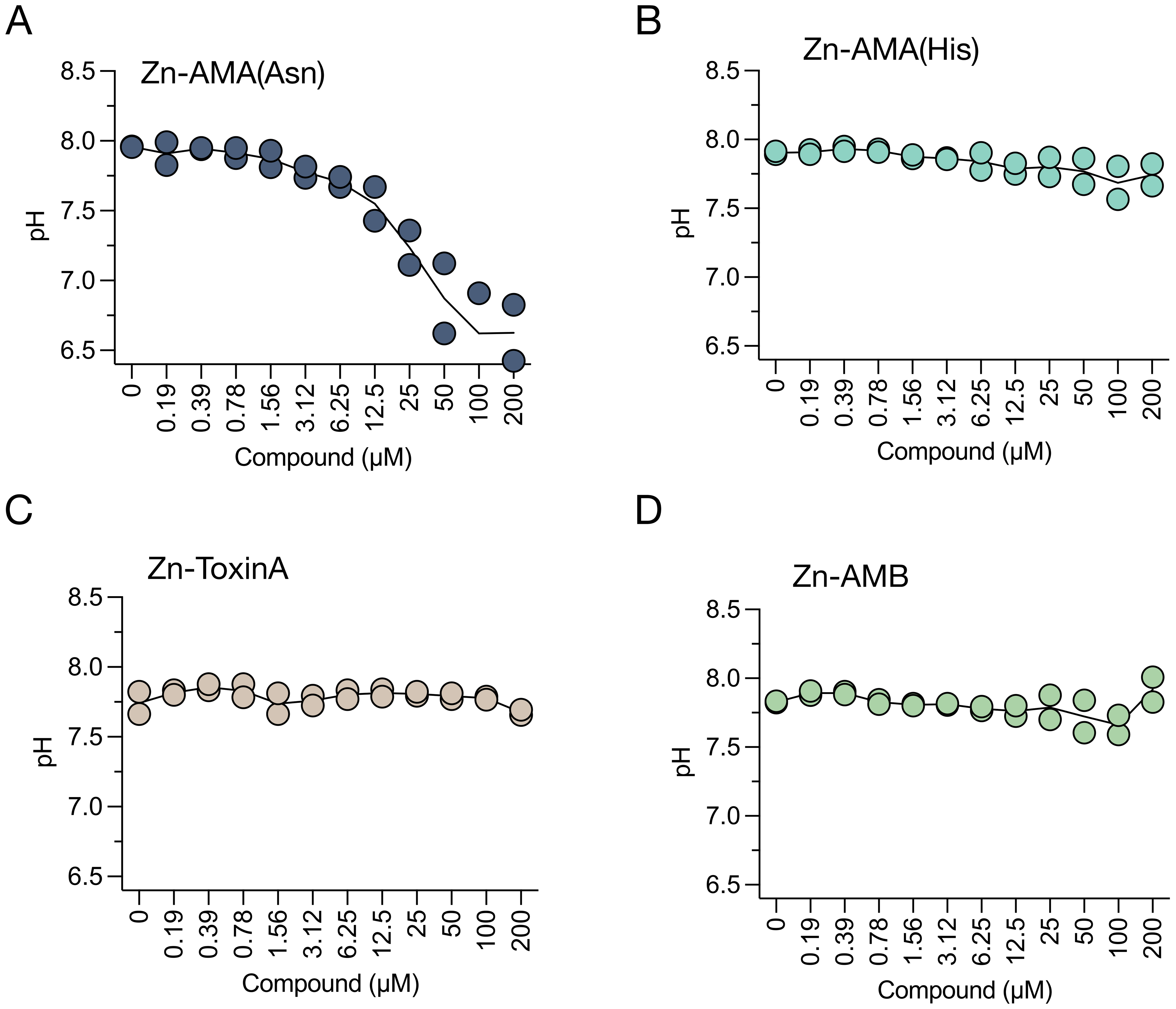

FIG S14 Structure-activity relationships of AMA analogs toward whole cell urease activity in *K. pneumoniae*. Dose-response curves of (A) Zn-AMA(Asn), (B) Zn-AMA(His), (C) Zn-toxin A, and Zn-AMB toward urease activity in *K. pneumoniae.* Urease activity was determined in artificial urine supplemented with 2-fold dilutions of AMA and its metal complexes at 37 ºC for 24 hours. Urease activity was measured as a change resulting from a pH change in the media with phenol red. Individual replicates are shown as colored circles with means connected with a black line.

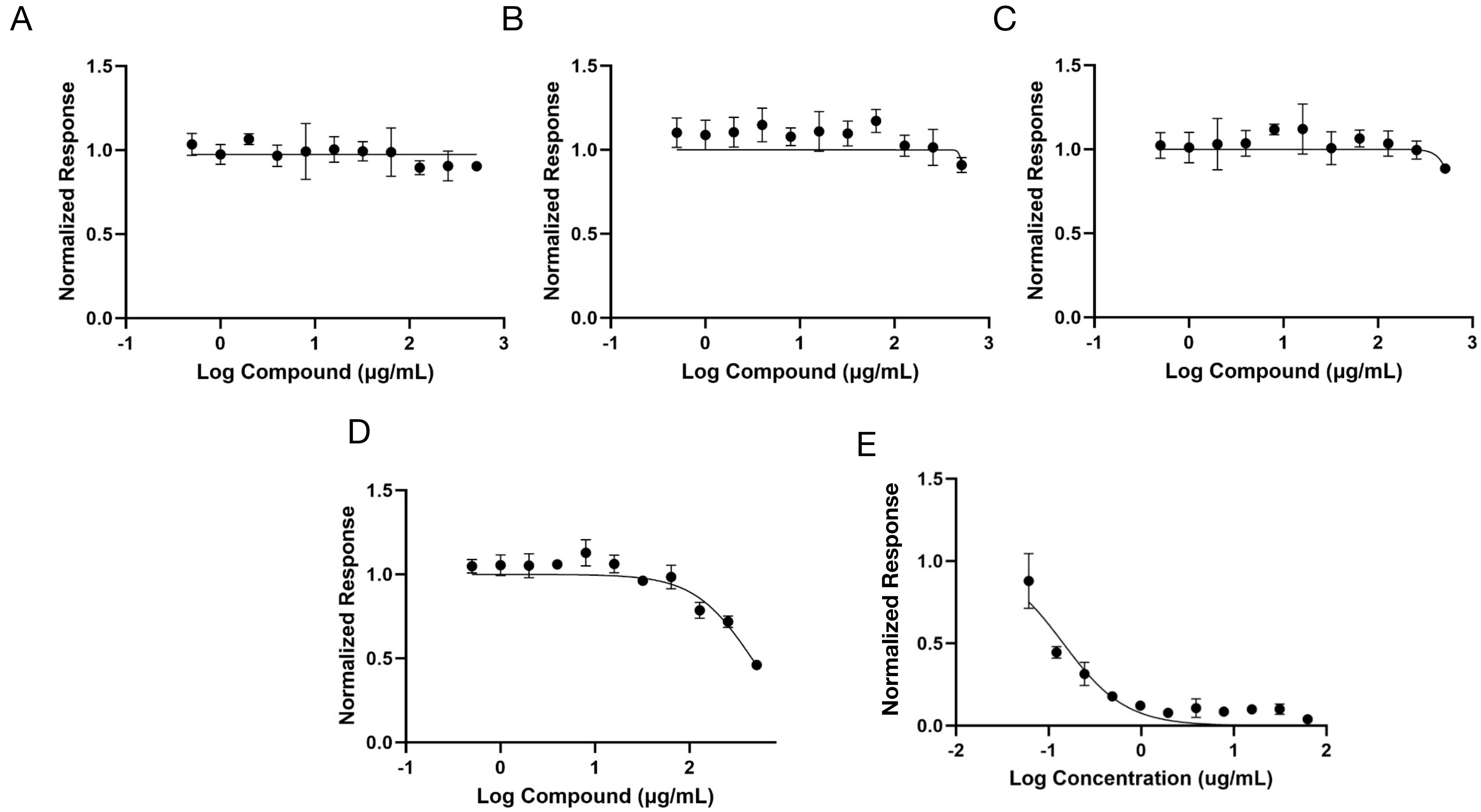

FIG S15 HEK293 cytotoxicity assays of AMA, Zn-AMA, Co-AMA, and Ni-AMA. Dose-response curves for (A) AMA, (B) Zn-AMA, (C) Ni-AMA, (D) Co-AMA, and (E) MG-132 positive control. The highest compound concentration for the AMA series was 512 μg/mL, which was diluted two-fold down to 0.5 μg/mL. The highest concentration for MG-132 was 62.5 μg/mL. HEK293 viability was measured with luminescence using CellTiter-Glo 2.0. The data represent the mean ± s.e. of triplicate experiments.

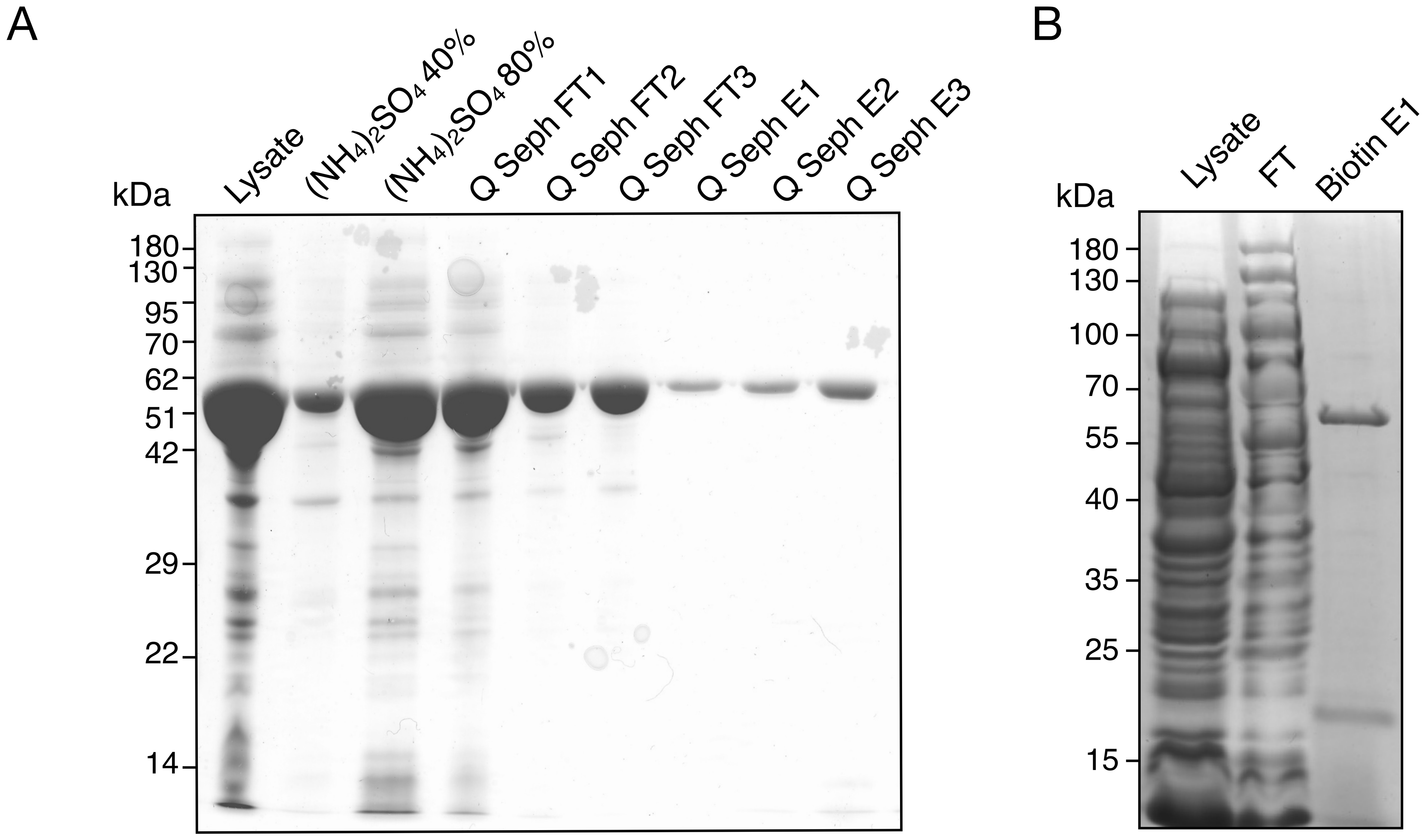

FIG S16. **Purification of NikA orthologs from *E. coli* and *K. pneumoniae.*** (A) SDS-PAGE analysis of *E. coli* NikA. The lanes labelled Q Seph E1 - E3 represent peak fractions eluting from a Q-Sepharose anion exchange column. (B) SDS-PAGE analysis of StrepII-tagged *K. pneumoniae* NikA. The lane labelled Biotin E1 represents the biotin elution from the StrepTrap XT column.

Table S1 NikA:Ni-AMA data collection and refinement statistics

	NikA:Ni-AMA (PDB ID: 8SPM)
**Data collection**	
Space group	$P4_122$
Cell dimensions	
a, b, c (Å)	66.42, 66.42, 267.77
α, β, γ (°)	90, 90, 90
Resolution (Å)	47.15 - 2.15 (2.23 - 2.15)
R_{merge} (%)	0.094 (4.45)
$CC_{1/2}$	0.999 (0.253)
I / σI	18.15 (0.77)
Completeness (%)	93.04 (74.89)
Multiplicity	14.1 (14.8)
**Refinement**	
Total No. reflections	473958 (48235)
No. unique reflections	33650 (3262)
R_{work} / R_{free}	0.189/0.242
No. atoms	
Protein	3954
Ligand	22
Water	102
B-factors	
Protein	60.6
Zn-AMA	50.79
Water	54.93
R.m.s. deviations	
Bond lengths (Å)	0.012
Bond angles (°)	1.19

*Data collection was performed using a single crystal.
Values in parentheses are for highest-resolution shell.
