# Supplementary material for: Targeting bacterial nickel transport with aspergillomarasmine A suppresses virulence-associated Ni-dependent enzymes
Source: Nat Commun. 2024 May 13;15:4036. doi: 10.1038/s41467-024-48232-1 (PMC11091072; doi:10.1038/s41467-024-48232-1)
Supplement: Supplementary file 3 — Reporting Summary [file 41467_2024_48232_MOESM3_ESM.pdf]

nature portfolio

Corresponding author(s): Gerard Wright

Last updated by author(s): 2023/12/06

Reporting Summary

Nature Portfolio wishes to improve the reproducibility of the work that we publish. This form provides structure for consistency and transparency in reporting. For further information on Nature Portfolio policies, see our Editorial Policies and the Editorial Policy Checklist.

Statistics

For all statistical analyses, confirm that the following items are present in the figure legend, table legend, main text, or Methods section.

n/a	Confirmed	
☐	☒	The exact sample size (*n*) for each experimental group/condition, given as a discrete number and unit of measurement
☐	☒	A statement on whether measurements were taken from distinct samples or whether the same sample was measured repeatedly
☒	☐	The statistical test(s) used AND whether they are one- or two-sided<br>*Only common tests should be described solely by name; describe more complex techniques in the Methods section.*
☒	☐	A description of all covariates tested
☒	☐	A description of any assumptions or corrections, such as tests of normality and adjustment for multiple comparisons
☐	☒	A full description of the statistical parameters including central tendency (e.g. means) or other basic estimates (e.g. regression coefficient) AND variation (e.g. standard deviation) or associated estimates of uncertainty (e.g. confidence intervals)
☐	☒	For null hypothesis testing, the test statistic (e.g. *F*, *t*, *r*) with confidence intervals, effect sizes, degrees of freedom and *P* value noted<br>*Give P values as exact values whenever suitable.*
☒	☐	For Bayesian analysis, information on the choice of priors and Markov chain Monte Carlo settings
☒	☐	For hierarchical and complex designs, identification of the appropriate level for tests and full reporting of outcomes
☒	☐	Estimates of effect sizes (e.g. Cohen's *d*, Pearson's *r*), indicating how they were calculated

Our web collection on statistics for biologists contains articles on many of the points above.

Software and code

Policy information about availability of computer code

Data collection	NikA data collection:<br>MxDC: https://michel4j.github.io/mxdc/
Data analysis	NikA structure data analysis:<br>XDS: http://xds.mpimf-heidelberg.mpg.de/<br>CCP4: https://ccp4.ac.uk/<br>Phenix: https://phenix-online.org<br>Coot: https://www2.mtc-lmb.cam.au.uk/personal/pemsley/coot<br>MolProbity: https://molprobity.biochem.duke.edu/<br>TLSMD: https://skuld.bmsc.washington.edu/~tlsmd/<br>GradeWebServer: https://grade.globalphasing.org/

For manuscripts utilizing custom algorithms or software that are central to the research but not yet described in published literature, software must be made available to editors and reviewers. We strongly encourage code deposition in a community repository (e.g. GitHub). See the Nature Portfolio guidelines for submitting code & software for further information.

Data

Policy information about availability of data

All manuscripts must include a data availability statement. This statement should provide the following information, where applicable:

- Accession codes, unique identifiers, or web links for publicly available datasets
- A description of any restrictions on data availability
- For clinical datasets or third party data, please ensure that the statement adheres to our policy

The coordinates and structure factors of NikA:Ni-AMA generated in this study are available at the PDB under the accession number 8SPM [https://doi.org/10.2210/pdb8SPM/pdb]. The previously published coordinates for NikA apoprotein, NikA:Fe-EDTA, NikA-Ni(L-His)2, and NikA:Ni-BTC are available at the PDB under the accession numbers, 1UIU [https://doi.org/10.2210/pdb1UIU/pdb], 1ZLQ [https://doi.org/10.2210/pdb1ZLQ/pdb], 4I8C [https://doi.org/10.2210/pdb4I8C/pdb], and 3DP8 [https://doi.org/10.2210/pdb3DP8/pdb], respectively. All other data are available upon request.

Research involving human participants, their data, or biological material

Policy information about studies with human participants or human data. See also policy information about sex, gender (identity/presentation), and sexual orientation and race, ethnicity and racism.

Reporting on sex and gender	Not applicable
Reporting on race, ethnicity, or other socially relevant groupings	Not applicable
Population characteristics	Not applicable
Recruitment	Not applicable
Ethics oversight	Not applicable

Note that full information on the approval of the study protocol must also be provided in the manuscript.

Field-specific reporting

Please select the one below that is the best fit for your research. If you are not sure, read the appropriate sections before making your selection.

☒ Life sciences ☐ Behavioural & social sciences ☐ Ecological, evolutionary & environmental sciences

For a reference copy of the document with all sections, see nature.com/documents/nr-reporting-summary-flat.pdf

Life sciences study design

All studies must disclose on these points even when the disclosure is negative.

Sample size	No sample size calculations were performed, and two replicates were performed to sufficiently capture variability in in vitro biochemical or microbial culture assays.
Data exclusions	No data was excluded from the analysis.
Replication	All experiments where summary values are reported were representative experiments of technical duplicate repeated independently at least twice. The exception being biofilm assays performed in quadruplicate, repeated independently at least twice.
Randomization	Galleria larvae were chosen randomly from a pool of live specimens and of similar size.
Blinding	No blinding was performed and was not necessary because the results were quantitative and not subjective. Blinding is not typical for these type of experiments.

Reporting for specific materials, systems and methods

We require information from authors about some types of materials, experimental systems and methods used in many studies. Here, indicate whether each material, system or method listed is relevant to your study. If you are not sure if a list item applies to your research, read the appropriate section before selecting a response.

Materials & experimental systems

n/a	Involved in the study
☐	☒ Antibodies
☐	☒ Eukaryotic cell lines
☐	☐ Palaeontology and archaeology
☐	☒ Animals and other organisms
☐	☐ Clinical data
☐	☐ Dual use research of concern
☐	☐ Plants

Methods

n/a	Involved in the study
☒	☐ ChIP-seq
☒	☐ Flow cytometry
☒	☐ MRI-based neuroimaging

Antibodies

Antibodies used	Mouse anti-FLAG(DYKDDDK)tag-HRP (Genscript, A01428.), Rabbit anti-OmpA (Cusabio, CSB-PA359226ZA01EOD), and Mouse anti-Rabbit-HRP (Cayman, 10004301).
Validation	All antibodies were confirmed by western blotting as reported by the manufacturer.

Eukaryotic cell lines

Policy information about cell lines and Sex and Gender in Research

Cell line source(s)	Human embryonic kidney cells 293 (HEK293) ATCC CRL-1573; generation 15
Authentication	Cells lines were not authenticated
Mycoplasma contamination	Cells were not tested for mycoplasma contamination
Commonly misidentified lines (See ICLAC register)	No commonly misidentified lines were used in this study

Palaeontology and Archaeology

Specimen provenance	Not applicable
Specimen deposition	Not applicable
Dating methods	Not applicable

☐ Tick this box to confirm that the raw and calibrated dates are available in the paper or in Supplementary Information.

Ethics oversight	Not applicable

Note that full information on the approval of the study protocol must also be provided in the manuscript.

Animals and other research organisms

Policy information about studies involving animals; ARRIVE guidelines recommended for reporting animal research, and Sex and Gender in Research

Laboratory animals	Galleria mellonella, 2 weeks old
Wild animals	No wild animals were used in this study.
Reporting on sex	Not applicable
Field-collected samples	No field-collected samples were used in this study
Ethics oversight	This study did not require ethics oversight.

Note that full information on the approval of the study protocol must also be provided in the manuscript.

Clinical data

Policy information about clinical studies

All manuscripts should comply with the ICMJEguidelines for publication of clinical research and a completedCONSORT checklist must be included with all submissions.

Clinical trial registration	Not applicable
Study protocol	Not applicable
Data collection	Not applicable
Outcomes	Not applicable

Dual use research of concern

Policy information about dual use research of concern

Hazards

Could the accidental, deliberate or reckless misuse of agents or technologies generated in the work, or the application of information presented in the manuscript, pose a threat to:

No	Yes	
☒	☐	Public health
☒	☐	National security
☒	☐	Crops and/or livestock
☒	☐	Ecosystems
☒	☐	Any other significant area

Experiments of concern

Does the work involve any of these experiments of concern:

No	Yes	
☒	☐	Demonstrate how to render a vaccine ineffective
☒	☐	Confer resistance to therapeutically useful antibiotics or antiviral agents
☒	☐	Enhance the virulence of a pathogen or render a nonpathogen virulent
☒	☐	Increase transmissibility of a pathogen
☒	☐	Alter the host range of a pathogen
☒	☐	Enable evasion of diagnostic/detection modalities
☒	☐	Enable the weaponization of a biological agent or toxin
☒	☐	Any other potentially harmful combination of experiments and agents

Plants

Seed stocks	Not applicable
Novel plant genotypes	Not applicable
Authentication	Not applicable
